# Prevalence and Burden of Disorders of Gut–Brain Interaction in Nigeria: Results From the Rome Foundation Global Epidemiology Study

**DOI:** 10.1111/nmo.70480

**Published:** 2026-11-02

**Authors:** Abdulsalam I. Aliyu, Tom van Gils, Jóhann P. Hreinsson, Mary Duguru, Pantong Davwar, Michael Okorie, David Nyam, Patience Omaiye, Crystal Umejiaku, Olive Obienu, Uchenna Ijoma, Mbang Kooffreh‐Ada, Kefas Zawaya, Charles Onyekwere, Adegboyega Akere, Shrikant I. Bangdiwala, Hans Törnblom, Olafur S. Palsson, Ami D. Sperber, Edith Okeke, Magnus Simrén

**Affiliations:** ^1^ Department of Molecular and Clinical Medicine, Institute of Medicine, Sahlgrenska Academy University of Gothenburg Gothenburg Sweden; ^2^ Department of Medicine Sahlgrenska University Hospital Gothenburg Västra Götaland Sweden; ^3^ Department of Medicine University of Jos, Jos University Teaching Hospital Jos Nigeria; ^4^ University of Nigeria Teaching Hospital Enugu Enugu Nigeria; ^5^ University of Calabar Teaching Hospital Calabar Cross River Nigeria; ^6^ Federal Teaching Hospital Gombe Gombe Nigeria; ^7^ Lagos State University Teaching Hospital Lagos Lagos Nigeria; ^8^ University College Hospital Ibadan Oyo Nigeria; ^9^ Department of Health Research Methods, Evidence and Impact, and Population Health Research Institute McMaster University Hamilton Ontario Canada; ^10^ Center for Functional GI & Motility Disorders University of North Carolina‐Chapel Hill Chapel Hill North Carolina USA; ^11^ Faculty of Health Sciences Ben‐Gurion University of the Negev Beer‐Sheva Israel

**Keywords:** burden, disorders of gut–brain interaction, functional dyspepsia, irritable bowel syndrome, Nigeria, prevalence

## Abstract

**Background:**

Data on the prevalence and impact of disorders of gut–brain interaction (DGBI) are lacking for most parts of Africa, including Nigeria, the country with the largest population on the continent. This study aimed to investigate the prevalence and burden of DGBI among adults in the Nigerian general population.

**Methods:**

Nigerian data from the Rome Foundation Global Epidemiology Study (RFGES) were obtained by trained interviewers using face‐to‐face household interviews. The questionnaire included the Rome IV diagnostic questionnaire to assess the presence of DGBI, psychological distress (PHQ‐4), non‐GI somatic symptom severity (PHQ‐12), mental and physical quality of life (PROMIS‐10), health care utilization, and dietary intake.

**Key Results:**

One thousand six hundred and thirty‐two individuals (48.6% females, mean age 44 (range 18–100) years) were sampled. The prevalence of any DGBI in Nigeria was 23.5% (95% CI: 21.5–25.6), with a higher prevalence in females (26.1%) than in males (21.0%). The highest prevalence of DGBI per gastrointestinal anatomical region was for bowel disorders (17.6%). The most prevalent individual DGBI diagnoses were functional dyspepsia (7.3%) and unspecified functional bowel disorder (6.6%). Compared to individuals without DGBI, individuals with DGBI were older, had reduced quality of life, increased psychological symptoms and associated healthcare utilization burden. There was a higher prevalence of any DGBI reported in the northern regions than in the southern regions.

**Conclusions:**

DGBI are prevalent and impactful in Nigeria, in line with studies from other parts of the world that utilized the face‐to‐face survey methodology. More studies are needed to further understand the distribution and impact of DGBI in Nigeria and other similar countries around the world where they are understudied.

## Key Points

- DGBI are prevalent and impactful in Nigeria, in line with studies from other similar countries around the world that utilized face‐to‐face survey methodology.
- Northern geopolitical zones of Nigeria have the highest burden of any DGBI prevalence.
- Having any DGBI was associated with older age, increased psychological symptoms, and increased healthcare utilization in the Nigerian adult population.

## Graphical abstract

The study provides the first comprehensive national population‐based prevalence of disorders of gut–brain interaction (DGBI) for Nigeria. We demonstrated that DGBI are prevalent and impactful in the Nigerian adult population and the northern region showed a higher burden of the DGBI.

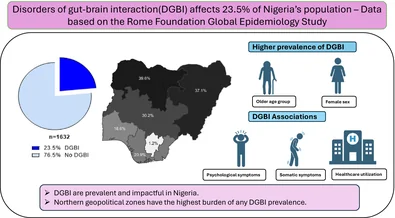

## 1 Introduction

Disorders of gut–brain interaction (DGBI) are prevalent, with a reported global prevalence of 40.3% [1] using an internet survey method. These disorders are defined based on the presence of various combinations of chronic or recurrent GI symptoms like pain, bloating, vomiting, or abnormal bowel habits in the absence of an organic disease and are often associated with impaired quality of life [2], psychological distress [3, 4] and increased healthcare burden [5, 6, 7].

Nigeria is the country with the largest population in the African continent and is a lower middle‐income country with a diverse cultural, ethnic, religious, and social class society. Its combination of low income and high population density has resulted in poor health outcomes, with the burden of non‐communicable diseases making up 27.7% of deaths in 2021 [8].

Like most non‐communicable diseases, data about the national prevalence of DGBI in Nigeria are lacking. The initial attempt to assess the prevalence of irritable bowel syndrome (IBS) dates back to 1988 when Atoba reported the prevalence of IBS among patients referred to a single tertiary center for bowel complaints [9]. Since then, most of the data regarding DGBI in Nigeria have focused either on a single DGBI (irritable bowel syndrome, functional diarrhea, or functional abdominal pain), the pediatric/adolescent age group, or a small section of the Nigerian population [10, 11, 12, 13, 14, 15, 16, 17]. This finding highlights the need for representational national prevalence data to understand the distribution and impact of DGBI in Nigeria, to help manage them better, and to develop future healthcare plans in the country.

The aim of this study was therefore to determine the prevalence and associated factors of DGBI in the general adult population of Nigeria.

## 2 Methods and Materials

### 2.1 Study Population

The study population was the subset of participants recruited in the Rome Foundation Global Epidemiology Study (RFGES) [1], for which global data have been published. In this global study, 33 countries were surveyed, 24 countries utilizing internet‐based surveys only, seven others including Nigeria used in‐person interviews only, whilst 2 countries used both internet and in‐person interviews (Turkey and China). A minimum of 2000 individuals was set as a target for each country. Pre‐defined demographic parameters included 50% females and 50% males; 50% rural and 50% urban populations; and an age distribution of 40% for 18–39 years, 40% for 40–64 years, and 20% for 65+ years. Ethics approval was obtained for the Nigerian study, and the study was conducted in accordance with the Helsinki declaration, after participants provided written informed consent to participate in the survey.

### 2.2 Data Collection and Questionnaires

Data were collected between November 2017 and May 2018 in all six Nigerian geopolitical zones, by trained local interviewers by means of a paper‐based survey booklet. Interviews were conducted in the houses of the study participants and questionnaires completed by the trained interviewers. The overall study design for the household surveys was described previously [18] and the participants were not informed that the aim of the study was to assess GI symptoms/DGBI, but instead, that this was a health‐related questionnaire. For convenience, our study adopted the facility and mapping of Removing the Mask on Hypertension (REMAH) study in Nigeria [19]. Households (survey participants) were randomly selected from two randomly selected communities (rural and urban) of a state from each of the six geopolitical zones (north‐central, north‐east, north‐west, south‐east, south‐south, and south‐west) using a multistage sampling technique. For this survey, the randomly selected states were Anambra, Akwa‐Ibom, Oyo, Zamfara, Gombe, and FCT‐Abuja.

The RFGES survey used the Rome IV diagnostic questionnaire (89 questions on gastrointestinal symptoms) [20]. It also used an 80‐item supplemental questionnaire on sociodemographic characteristics, medical and health history, comorbid symptoms and conditions, GI infections, healthcare utilization, current living conditions, psychosocial variables, diet, and religious or spiritual affiliation. It also included validated questionnaires that were embedded in the full study questionnaire, such as the Patient Health Questionnaire‐12 (PHQ‐12) [21] with the question about menstruation excluded. The PHQ‐12 assesses how bothersome 12 somatic non‐GI symptoms were in the past month, each scored as 0—not bothered at all, 1—bothered a little, and 2—bothered a lot with a maximum possible score of 22. Psychological symptoms were assessed using the Patient Health Questionnaire‐4 (PHQ‐4) [22], which is comprised of four components assessing the frequency of symptoms of anxiety and depression over the last 2 weeks, each scored as 0—not at all, 1—several days, 2—more than half of the days, or 3—nearly every day. Health‐related QoL was assessed using the Patient‐Reported Outcomes Measurement Information System (PROMIS) Global‐10 questionnaire, which scores general domains of health and functioning. The questionnaire yields two global domain summary scores, physical and mental QoL. A higher score indicates a better QoL, with a range of 4–20 per domain [23]. The Work Productivity and Activity Impairment (WPAI) questionnaire assesses four domains over the last 7 days, which are (i) absenteeism, absence from work due to health issues; (ii) presenteeism, reduced work productivity while at work due to health issues; (iii) overall work impairment, a combination of absenteeism and presenteeism; and (iv) activity impairment, in activity and/or productivity outside of work due to health issues. Scores are calculated from 0% to 100%, with a greater percentage indicating greater WPAI [24]. Participants also completed food frequency questionnaires where they were asked to record on average how many days a week they consumed 5 food groups (fruit, vegetables/legumes, milk products, cereals, tubers) and 8 other types of food (rice, bread, pasta, meat, fish, eggs, acha, and awara).

### 2.3 Data Handling

Participants who otherwise met Rome IV DGBI criteria were excluded from the DGBI group if they self‐reported a medical history that could represent organic or structural reasons for the symptoms. Those participants reporting a physician‐based diagnosis of coeliac disease, GI cancer, or inflammatory bowel disease (Crohn's disease or ulcerative colitis) were excluded from all Rome IV DGBI diagnoses. Subjects with a history of peptic ulcer disease were excluded from esophageal, gastroduodenal, and biliary diagnoses but included in bowel and anorectal disorders. Those participants who reported diverticulitis or bowel resection were excluded from bowel and anorectal disorders but included in esophageal, gastroduodenal, or biliary diagnoses. Those who were excluded from the DGBI group remained in the denominator.

Missing and inconsistent questionnaire responses were handled using deterministic rules based on the skip structure of the Rome IV Diagnostic Questionnaire. If a stem symptom question indicated absence of the symptom, any dependent follow‐up responses were re‐coded as missing. If a stem question was missing but the corresponding follow‐up questions had been completed, the stem response was inferred by assigning the modal non‐zero response for that item. If both the stem question and relevant follow‐up information were missing, the missing values were retained. Additional nested skip‐pattern corrections were applied where applicable. No multiple imputations were performed.

Complete data to assess the presence or absence of all 22 DGBI were available for 1632 individuals (77%). These individuals were used to assess the prevalence of fulfilling the criteria for “any DGBI”, that is, at least one DGBI. For individual DGBI and DGBI per anatomical region, all individuals with complete data to assess the presence or absence of that specific DGBI were used.

### 2.4 Healthcare Utilization

Health care utilization was assessed by asking about the frequency of doctor visits for any health problem ranging from “once a month or more,” “a few times a year,” “once a year,” “less than once a year” to “never”. Participants also reported their usual source of care when sick or injured (western style medicine, traditional/folk healer, both, or neither) and whether they could access a doctor when needed (“yes, easily,” “yes, but it would be difficult,” or “no”).

### 2.5 Statistical Analysis

Prevalence rates were calculated for individual DGBI (national and regional) and for defined gastrointestinal (GI) anatomical regions and reported as percentages with 95% confidence intervals (95% CI). Categorial variables were presented as proportions (%) with 95% CI and continuous variables as mean (95% CI). Odds ratios (OR) and standardized mean differences (SMD)/Cohen's d including 95% CI were used to estimate the magnitude of difference between the groups for categorical and continuous variables, respectively. Standardized mean differences of 0.2–0.5 were considered small, 0.5–0.8 as medium, and values > 0.8 as large [25, 26]. Statistical analyses were undertaken using SPSS software (Version 29.0, SPSS Inc., Chicago, USA) and figures created using GraphPad Prism version 11.0.0 for Windows, GraphPad Software, San Diego California USA, www.graphpad.com.

## 3 Results

### 3.1 Study Population

In total 2118 individuals were registered in the survey, and 1632 participants were analyzed for the prevalence of any DGBI (48.6% females, mean age 44.4 (95% CI; 43.6–45.2) years). Their other sociodemographic characteristics are presented in Table 1.

**TABLE 1:** Socio‐demographic characteristics of participants.

Sociodemographic characteristics	Any DGBI (*n* = 383)	No DGBI (*n* = 1249)	SMD/OR (95% CI)
Age, years	46.2 (44.5–47.9)	43.9 (43.0–44.8)	0.1 (0.0–0.3)
18–39 years % (95% CI)	36.6 (31.9–41.5)	40.4 (37.7–43.2)	1.5 (1.1–2.0)^‡^
40–64 years % (95% CI)	40.2 (35.4–45.2)	42.0 (39.3–44.8)	1.4 (1.0–1.9)^‡^
> 65 years % (95% CI)	23.2 (19.3–27.7)	17.5 (15.5–19.7)	Reference
Females (%)	54.0 (49.0–59.0)	46.9 (44.2–49.7)	1.3 (1.1–1.8)^‡^
BMI (kg/m^2^)	23.7 (23.2–24.2)	25.5 (25.2–25.8)	−0.4 (−0.5 to −0.2)
Education duration (years)	8.5 (7.6–8.9)	11.5 (11.2–11.8)	−0.6 (−0.7 to −0.5)
Employed (%)	23.1 (19.0–27.7)	20.6 (18.4–22.9)	1.2 (0.9–1.6)^‡^
Living conditions			
Rural (%)	58.2 (53.1–63.0)	44.9 (42.2–47.7)	1.7 (1.3–2.1)^‡^
Religion			
Christianity (%)	52.6 (47.3–57.8)	69.0 (66.2–71.6)	Reference
Islam (%)	39.6 (34.6–44.8)	25.5 (23.0–28.1)	2.0 (1.6–2.6)^‡^
Others (%)	2.6 (1.3–4.9)	2.0 (1.3–3.0)	1.7 (0.8–3.7)^‡^
No response (%)	5.2 (3.3–8.1)	3.5 (2.6–4.8)	1.9 (1.1–3.5)^‡^

*Note:* Data are presented as means or proportions with 95% confidence interval (CI). Comparisons were using odds ratio (OR) ‡ or Standardized mean difference (SMD) with 95% CI. Body mass index (BMI).

### 3.2 Prevalence of DGBI in Nigeria

The overall prevalence of any DGBI in Nigeria was 23.5% (95% CI; 21.5–25.6) (Figure 1), with a higher prevalence in females (26.1%) than in males (21.0%) (Table 2). The highest prevalence of DGBI per GI anatomical region was in bowel disorders (17.6%), followed by gastroduodenal disorders (9.0%). Esophageal disorders had the lowest prevalence (3.6%). The most prevalent individual DGBI diagnoses in decreasing order were functional dyspepsia (7.3%), unspecified functional bowel disorder (6.6%), proctalgia fugax (4.6%), functional constipation (4.2%), and irritable bowel syndrome (3.0%). Among individuals with any DGBI, 12.0% had one DGBI diagnosis, 7.5% had two diagnoses, 2.4% had three diagnoses, and 1.6% had four or more DGBI diagnoses. Age‐specific prevalence was highest in the ≥ 65 years age group (28.9%) and lowest in the 18–39 years age group (21.7%).

**FIGURE 1:**
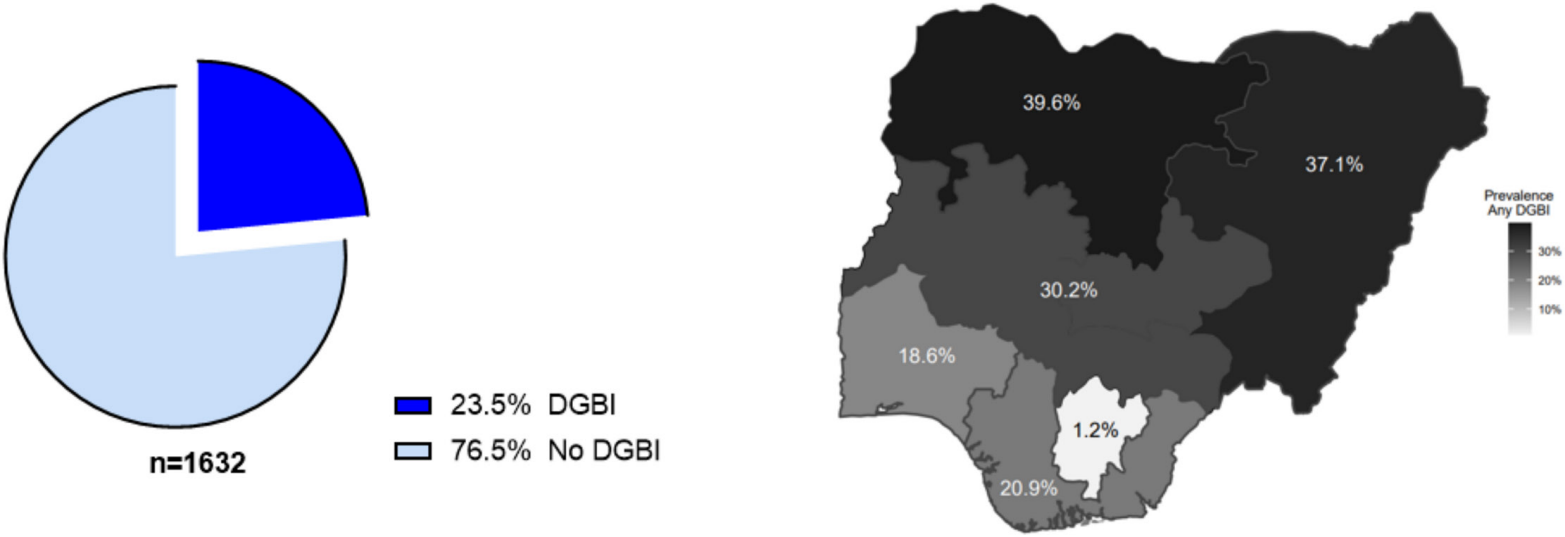
Prevalence of any DGBI in the Nigerian population according to the six geopolitical zones. Percentages are proportions of individuals with any DGBI from the population that responded to the survey.

**TABLE 2:** Prevalence of disorders of gut–brain interactions (DGBI) in Nigeria.

DGBI	Overall prevalence (*n* = 1632)	Sex		Age group (years)		
		Male (*n* = 839)	Female (*n* = 793)	18–39 (*n* = 645)	40–64 (*n* = 679)	≥ 65 (*n* = 308)
Any DGBI	**23.5 (21.5–25.6)**	**21.0 (18.4–23.9)**	**26.1 (23.2–29.3)**	**21.7 (18.7–25.1)**	**22.7 (19.7–26.0)**	**28.9 (24.1–34.2)**
1 DGBI	12.0 (10.5–13.7)	11.3 (9.3–13.7)	12.7 (10.6–15.2)	12.7 (10.4–15.5)	10.8 (8.6–13.3)	13.3 (9.9–17.6)
2 DGBI	7.5 (6.3–8.9)	7.3 (5.7–9.2)	7.7 (6.0–9.8)	6.0 (4.4–8.2)	8.8 (6.9–11.2)	7.5 (5.0–11.0)
3 DGBI	2.4 (1.7–3.3)	1.8 (1.1–3.0)	3.0 (2.0–4.5)	2.0 (1.1–3.5)	1.6 (0.9–2.9)	4.9 (2.9–7.9)
4+ DGBI	1.6 (1.1–2.3)	0.6 (0.2–1.4)	1.3 (0.7–2.3)	0.5 (0.0–1.4)	1.2 (0.6–2.4)	1.3 (0.0–3.4)
Esophageal disorders	**3.6 (2.9–4.6)**	**3.0 (2.1–4.2)**	**4.3 (3.2–5.7)**	**3.1 (2.1–4.6)**	**2.7 (1.8–4.1)**	**6.6 (4.5–9.6)**
Functional heart burn	0.6 (0.3–1.1)	0.4 (0.1–1.1)	0.9 (0.4–1.9)	0.5 (0.1–1.4)	0.4 (0.1–1.4)	1.3 (0.4–3.4)
Functional chest pain	0.7 (0.4–1.2)	0.7 (0.3–1.6)	0.6 (0.2–1.5)	0.8 (0.3–1.9)	0.3 (0.0–1.1)	1.3 (0.4–3.4)
Reflux Hypersensitivity	1.1 (0.7–1.7)	0.7 (0.3–1.6)	1.5 (0.8–2.7)	1.1 (0.5–2.3)	0.7 (0.3–1.8)	1.9 (0.8–4.3)
Globus	0.2 (0.0–0.6)	0.2 (0.0–0.9)	0.1 (0.0–0.8)	0.2 (0.0–1.0)	0.3 (0.0–1.1)	0.0 (0.0–1.5)
Functional dysphagia	1.0 (0.6–1.7)	0.7 (0.3–1.4)	1.4 (0.7–2.5)	0.9 (0.4–2.1)	0.6 (0.2–1.6)	2.3 (1.0–4.7)
Gastroduodenal disorders	**9.0 (7.8–10.4)**	**7.9 (6.3–9.7)**	**10.2 (8.5–12.3)**	**8.6 (6.8–10.8)**	**6.5 (5.0–8.5)**	**15.2 (11.9–19.1)**
Functional dyspepsia	7.3 (6.2–8.5)	6.0 (4.7–7.6)	7.9 (6.4–9.7)	5.9 (4.5–7.7)	5.3 (4.0–7.1)	12.5 (9.6–16.1)
Post‐prandial distress syndrome	4.0 (3.2–5.0)	3.7 (2.7–5.0)	4.2 (3.1–5.6)	2.9 (2.0–4.3)	2.9 (2.0–4.3)	8.1 (5.8–11.2)
Epigastric pain syndrome	3.5 (2.7–4.3)	2.5 (1.7–3.7)	4.2 (3.1–5.6)	3.1 (2.1–4.5)	2.6 (1.7–3.9)	5.7 (3.8–8.4)
Belching disorder	1.0 (0.6–1.5)	0.8 (0.4–1.5)	1.2 (0.7–2.1)	1.1 (0.5–2.0)	0.8 (0.4–1.7)	1.2 (0.4–2.9)
Rumination syndrome	0.5 (0.2–0.9)	0.3 (0.1–0.9)	0.7 (0.3–1.4)	0.5 (0.1–1.2)	0.3 (0.1–1.1)	0.7 (0.1–2.2)
Chronic nausea and vomiting syndrome	0.3 (0.1–0.7)	0.1 (0.0–0.1)	0.6 (0.2–1.3)	0.5 (0.1–1.2)	0.2 (0.0–0.9)	0.2 (0.0–1.5)
Cyclic vomiting syndrome	0.5 (0.2–0.9)	0.4 (0.1–1.0)	0.6 (0.2–1.3)	0.5 (0.1–1.2)	0.5 (0.1–1.2)	0.5 (0.0–1.9)
Cannabinoid hyperemesis syndrome	0.0 (0.0–0.2)	0.0 (0.0–0.4)	0.0 (0.0–0.4)	0.0 (0.0–0.5)	0.0 (0.0–0.5)	0.0 (0.0–1.1)
Bowel disorders	**17.6 (16.0–19.4)**	**14.8 (12.7–17.2)**	**20.4 (18.0–23.1)**	**15.9 (13.5–18.7)**	**17.4 (14.9–20.3)**	**21.5 (17.6–26.0)**
Irritable bowel syndrome (IBS)	3.0 (2.3–3.8)	2.0 (1.3–3.0)	3.9 (2.9–5.3)	2.9 (2.0–4.3)	2.3 (1.5–3.6)	4.2 (2.6–6.6)
IBS‐C	0.8 (0.5–1.3)	0.3 (0.1–0.9)	1.3 (0.8–2.3)	0.5 (0.1–1.3)	0.7 (0.3–1.6)	1.7 (0.8–3.6)
IBS‐D	1.1 (0.8–1.7)	1.0 (0.6–1.9)	1.2 (0.7–2.1)	1.4 (0.8–2.5)	0.7 (0.3–1.6)	1.5 (0.6–3.3)
IBS‐M	0.6 (0.3–1.0)	0.1 (0.0–0.6)	1.1 (0.6–1.9)	0.4 (0.1–1.1)	0.6 (0.2–1.4)	1.0 (0.3–2.6)
IBS‐U	0.4 (0.2–0.8)	0.6 (0.2–1.3)	0.2 (0.0–0.7)	0.6 (0.2–1.4)	0.4 (0.1–1.1)	0.0 (0.0–1.1)
Functional constipation	4.2 (3.4–5.2)	3.8 (2.8–5.2)	4.2 (3.1–5.6)	3.8 (2.7–5.3)	4.3 (3.1–5.9)	3.9 (2.4–6.3)
Opioid induced constipation	1.8 (1.2–2.4)	1.4 (0.8–2.3)	2.0 (1.3–3.0)	0.9 (0.4–1.9)	2.1 (1.3–3.3)	2.5 (1.3–4.5)
Unspecified functional bowel disorder	6.6 (5.6–7.9)	4.7 (3.6–6.1)	7.5 (6.1–9.3)	5.9 (4.5–7.7)	5.8 (4.4–7.6)	7.1 (5.0–10.1)
Functional diarrhea	1.0 (0.7–1.6)	0.9 (0.5–1.7)	1.1 (0.6–2.0)	0.7 (0.3–1.6)	0.9 (0.4–1.9)	2.0 (0.9–3.9)
Functional bloating	0.4 (0.2–0.8)	0.4 (0.1–1.0)	0.4 (0.1–1.0)	0.6 (0.2–1.4)	0.2 (0.0–0.9)	0.2 (0.0–1.5)
Anorectal disorders	**7.7 (6.7–9.0)**	**5.7 (4.4–7.3)**	**9.9 (8.2–11.9)**	**6.6 (5.1–8.5)**	**8.1 (6.4–10.2)**	**9.4 (6.9–12.7)**
Fecal incontinence	0.8 (0.4–1.2)	0.4 (0.0–0.6)	1.1 (0.6–2.0)	0.7 (0.3–1.6)	0.5 (0.1–1.2)	1.5 (0.6–3.3)
Levator ani syndrome	2.4 (1.8–3.2)	1.1 (0.6–2.0)	3.6 (2.6–4.9)	2.0 (1.2–3.2)	2.6 (1.7–3.9)	2.7 (1.5–4.8)
Proctalgia fugax	4.6 (3.8–5.6)	4.1 (3.1–5.5)	4.9 (3.8–6.4)	3.8 (2.7–5.3)	4.9 (3.6–6.5)	5.4 (3.6–8.1)
Others						
Centrally mediated abdominal pain	0.2 (0.1–0.6)	0.1 (0.0–0.6)	0.4 (0.1–1.0)	0.2 (0.0–0.9)	0.2 (0.0–0.9)	0.2 (0.0–1.5)
Functional biliary pain	0.1 (0.0–0.3)	0.0 (0.0–0.4)	0.1 (0.0–0.6)	0.0 (0.0–0.5)	0.0 (0.0–0.5)	0.2 (0.0–1.5)

*Note:* Data are presented as % with 95% confidence interval (CI). Disorders of gut‐brain interactions (DGBI); irritable bowel syndrome (IBS); constipation predominant IBS (IBS‐C); diarrhea predominant IBS (IBS‐D); mixed type IBS (IBS) and Unclassified IBS (IBS‐U). The prevalences of any DGBI and the DGBI per anatomical regions are bold to help identify them within the large table.

Female sex, lower BMI, older age, livingin rural areas, and fewer years of education were associated with having a DGBI (Table 1). Christianity and Islam were the predominant religions among the survey participants, with half of the individuals with any DGBI practicing Christianity and nearly 40% practicing Islam. The employment rate among the study participants was generally low, with only 23.1% of individuals with DGBI employed and 20.6% of those without DGBI, a finding consistent with a high level of unemployment in Nigeria, in general.

### 3.3 Regional Prevalence of DGBI in Nigeria

A north‐south gradient was observed (Figure 1) with the highest prevalence of any DGBI seen in the north‐west region (39.6%), followed by north‐east (37.1%), north‐central (30.2%), south‐south (20.9%), south‐west (18.6%), and south‐east (1.2%). Functional dyspepsia (11.7%) and IBS (6.9%) were the most prevalent DGBI in the north‐west region, whilst unspecified functional bowel disorder (11.6%) and functional constipation (8.2%) were most prevalent in the north‐east (Table 3).

**TABLE 3:** Characteristics of participants for each region and most prevalent individual DGBI and DGBI by GI anatomical regions across the six geopolitical regions in Nigeria.

Characteristics and DGBI diagnosis	North‐central (*n* = 222)	North‐east (*n* = 267)	North‐west (*n* = 265)	South‐east (*n* = 332)	South‐south (*n* = 277)	South‐west (*n* = 242)
Age groups (years)						
18–39 *n* (%)	89 (40.1%)	104 (39.0%)	112 (42.3%)	125 (37.7%)	100 (36.1%)	101 (41.75%)
40–64 *n* (%)	101 (45.5%)	112 (41.9%)	103 (38.9%)	141 (42.5%)	114 (41.2%)	96 (39.7%)
≥ 65 *n* (%)	32 (14.4%)	51 (19.1%)	50 (18.9%)	66 (19.9%)	63 (22.7%)	45 (18.6%)
Sex (*n* = 1605)						
Female *n* (%)	99 (44.6%)	120 (44.9%)	136 (51.3%)	167 (50.3%)	145 (52.3%)	117 (48.3%)
Living conditions (*n* = 1586)	220	266	255	329	274	242
Rural *n* (%)	123 (55.9%)	132 (49.6%)	126 (49.4%)	131 (39.8%)	143 (52.2%)	115 (47.5%)
Education duration (years)	10.6 (10.0–11.3)	9.6 (8.8–10.3)	7.1 (6.3–8.0)	13.1 (12.5–13.6)	11.0 (10.3–11.6)	13.0 (12.4–13.7)
Individual DGBI diagnosis						
Functional dyspepsia	11.0 (8.2–14.8)	6.8 (4.6–10.0)	11.7 (8.8–15.6)	0.6 (0.0–2.2)	6.1 (3.9–9.3)	5.5 (3.5–8.5)
Unspecified functional bowel disorder	7.9 (5.5–11.3)	11.6 (8.7–15.4)	10.6 (7.8–14.3)	0.6 (0.0–2.2)	2.8 (1.4–5.2)	3.5 (1.9–6.1)
Proctalgia fugax	2.8 (1.5–5.2)	9.6 (6.9–13.2)	9.7 (7.0–13.3)	0.0 (0.0–1.3)	3.1 (1.6–5.6)	1.7 (0.7–3.8)
Functional constipation	3.1 (1.7–5.6)	8.2 (5.7–11.6)	6.9 (4.6–10.1)	0.0 (0.0–1.3)	3.4 (1.8–6.0)	2.3 (1.1–4.6)
Irritable bowel syndrome	4.5 (2.8–7.3)	3.7 (2.1–6.3)	6.9 (4.6–10.1)	0.3 (0.0–1.7)	1.2 (0.3–3.1)	1.2 (0.4–3.2)
DGBI by anatomical regions						
Esophageal disorders	8.7 (6.1–12.3)	4.4 (2.6–7.2)	3.8 (2.2–6.5)	0.3 (0.0–1.8)	3.1 (1.6–5.7)	2.1 (0.9–4.3)
Gastroduodenal disorders	16.3 (12.6–20.8)	7.8 (5.3–11.3)	16.1 (12.4–20.6)	0.6 (0.0–2.2)	7.0 (4.6–10.5)	7.5 (5.0–10.9)
Bowel disorders	22.0 (17.7–27.0)	30.9 (26.2–36.1)	32.3 (27.3–37.8)	0.9 (0.2–2.7)	9.6 (6.8–13.5)	11.2 (8.0–15.5)
Anorectal disorders	9.0 (6.3–12.5)	12.2 (9.2–16.2)	13.8 (10.5–17.9)	0.3 (0.0–1.8)	8.1 (5.6–11.7)	3.4 (1.8–6.0)

*Note:* Disorders of gut‐brain interactions (DGBI). Data presented are proportions (%) or means with 95% confidence interval (CI) where applicable for each geopolitical region.

### 3.4 Association of Having Any DGBI With Health Care Utilization, Psychological, Non‐GI Somatic Symptoms, and Work Productivity and Activity Impairment

A higher rate of health care utilization was reported among individuals with any DGBI compared to those without (Table 4). The proportion of individuals with any DGBI who visited a doctor due to bowel problems (22.1%) was higher than those with no DGBI (9.6%). Similarly, the proportion of individuals with any DGBI (14.0%) having ≥ 1 doctor visits/month for any health problem was higher than those with no DGBI (8.7%). About 62% of individuals with any DGBI vs. 43% of those with no DGBI sought medical services from a western‐style trained doctor while individuals with any DGBI (3.4%) vs. no DGBI (1.3%) sought medical services from a traditional or folk healer. A lower proportion of individuals with any DGBI (74.9%) reported that they could visit doctors easily if they needed it than those with no DGBI (85.7%) and a higher proportion of those that reported not being able to visit a doctor if they needed it among those with any DGBI (5.6%) than those with no DGBI (2.8%). About 7.7% of individuals with any DGBI self‐reported to have had an acute enteritis immediately prior to having their bothersome symptoms.

**TABLE 4:** Comparison of psychological, non‐GI somatic, quality of life, and healthcare utilization factors among individuals with and without DGBI.

Characteristics	Any DGBI (*n* = 383)	No DGBI (*n* = 1249)	SMD/OR (95% CI)
Psychological symptoms (PHQ‐4)	2.8 (2.5–3.1)	1.3 (1.2–1.5)	0.6 (0.5–0.7)
Non‐GI somatic symptoms (PHQ‐12)	5.9 (5.5–6.3)	2.9 (2.7–3.0)	1.0 (0.8–1.1)
Physical QOL (PROMIS‐10)	13.8 (13.5–14.1)	15.7 (15.6–15.9)	−0.7 (−0.8 to 0.6)
Mental QOL (PROMIS‐10)	13.1 (12.8–13.4)	14.6 (14.4–14.7)	−0.5 (−0.7 to 0.4)
Absenteeism (WPAI)	14.0 (8.2–19.0)	4.4 (2.6–6.3)	0.5 (0.3–0.8)
Presenteeism (WPAI)	7.0 (3.6–10.5)	3.9 (2.5–5.2)	0.3 (0.01–0.5)
Work impairment (WPAI)	18.0 (12.2–23.8)	7.0 (4.7–9.3)	0.6 (0.3–0.9)
Activity impairment (WPAI)	15.3 (12.9–17.7)	4.6 (3.8–5.4)	0.6 (0.5–0.8)
Visited a doctor for bowel problem (%)	22.1 (18.1–26.6)	9.6 (8.1–11.4)	2.7 (2.0–3.6)^‡^
How often do you go to a doctor for your health?			
Once a month or more (%)	14.0 (10.9–17.9)	8.7 (7.3–10.4)	1.5 (0.9–2.4)^‡^
A few times a year (%)	46.6 (41.6–51.6)	34.4 (31.8–37.1)	1.2 (0.8–1.8)
Once a year (%)	13.0 (9.9–16.7)	22.0 (19.7–24.3)	0.5 (0.3–0.9)
Less than once a year (%)	15.3 (12.0–19.3)	24.8 (22.5–27.3)	0.6 (0.4–0.9)
Never (%)	11.1 (8.3–14.7)	10.2 (8.6–12.0)	Reference
If you are sick or injured, where do you seek help?			
From a doctor trained in western style medicine (%)	61.9 (56.9–66.6)	76.2 (73.7–78.5)	Reference
From a traditional or folk healer (%)	3.4 (1.9–5.8)	3.5 (2.6–4.7)	1.2 (0.6–2.2)
Both (%)	29.5 (25.2–34.3)	15.8 (13.8–17.9)	2.3 (1.8–3.0)
Neither (%)	5.2 (3.4–8.0)	4.5 (3.5–5.8)	1.4 (0.8–2.4)
Would you be able to visit doctor if you really needed to do so?			
Yes, easily (%)	74.9 (70.3–79.0)	85.7 (83.6–87.5)	0.4 (0.3–0.8)
Yes, but it would be difficult (%)	19.6 (15.9–23.9)	11.5 (9.9–13.4)	0.9 (0.5–1.6)
No (%)	5.6 (3.6–8.4)	2.8 (2.0–3.9)	Reference

*Note:* Data are presented as means or proportions with 95% CI. Comparisons were using odds ratio (OR) or standardized mean difference (SMD) with 95% CI. Patient Health Questionnaire‐4 (PHQ‐4); Patient Health Questionnaire‐12 (PHQ‐12); Patient‐ Reported Outcomes Measurement Information System (PROMIS)‐10; Work Productivity and Activity Impairment (WPAI).

^‡^ to indicate the value is for odds ratio.

Individuals with any DGBI, compared to those with no DGBI (Table 4), had more psychological (combined anxiety and depression) symptoms (PHQ‐4; 2.8 vs. 1.3) and a higher non‐GI somatic symptom severity (PHQ‐12; 5.9 vs. 2.9). Individuals with any DGBI had both lower physical and mental QOL (PROMIS‐10; 13.8 and 13.1) than those with no DGBI (PROMIS‐10; 15.7 and 14.6). Higher degrees of absenteeism, presenteeism, overall work impairment and activity impairment were observed in individuals with any DGBI compared to those with no DGBI.

The regional differences in prevalence are supported by the higher severity of non‐GI somatic symptoms in the north‐central region (PHQ‐12 6.6 (95% CI: 5.9–7.4)) vs. south‐west region (5.5 (95% CI: 4.4–6.6)) and psychological symptoms in the north‐western (PHQ‐4 3.4 (95% CI: 2.9–4.0)) vs. the south‐western region (1.5 (95% CI: 0.8–2.2)) among individuals with DGBI (Table 5). Among individuals with any DGBI from the six geopolitical zones, the highest proportion of doctors' visits due to health problems was seen in the south‐west region and the lowest in the north‐west region despite having the largest prevalence (Table 5). The number of years spent on education was higher in the southern regions compared to the northern regions (Table 3).

**TABLE 5:** Factors associated with having any DGBI across the geopolitical zones.

Geopolitical zone	Doctor's visit (%)	Psychological symptoms (PHQ‐4)	Non‐GI somatic symptoms (PHQ‐12)	Mental QOL (PROMIS‐10)	Physical QOL (PROMIS‐10)
North‐central	13.4 (7.0–23.8)	2.2 (1.5–2.9)	6.0 (5.0–7.1)	12.9 (12.4–13.4)	14.1 (13.4–14.7)
North‐east	16.2 (10.1–24.8)	3.2 (2.5–3.9)	6.6 (5.9–7.4)	12.5 (12.0–13.1)	13.3 (12.7–13.9)
North‐west	11.8 (6.7–19.6)	3.4 (2.9–4.0)	5.4 (4.7–6.0)	13.8 (13.4–14.2)	14.3 (13.8–14.9)
South‐south	12.1 (5.7–23.2)	2.4 (1.9–2.9)	5.7 (4.6–6.7)	11.2 (10.7–11.8)	11.8 (11.1–12.4)
South‐west	20.0 (10.9–34.7)	1.5 (0.8–2.2)	5.5 (4.4–6.6)	15.3 (14.6–16.0)	15.6 (14.9–16.3)

*Note:* Data are presented as means and proportions (%) with their 95% confidence interval (CI) for only participants with any DGBI from the geopolitical zones of Nigeria.

### 3.5 Dietary Intake Pattern Among Individuals With Versus Without a DGBI (Table 6)

**TABLE 6:** Dietary intake pattern in individuals with vs. without DGBI.

Food items (mean number of days/week)	Any DGBI (*n* = 383)	No DGBI (*n* = 1249)	SMD (95% CI)
Milk products	2.1 (1.9–2.4)	2.6 (2.5–2.8)	−0.2 (−0.3 to –0.1)
Meat from animals	3.3 (3.1–3.6)	4.3 (4.2–4.5)	−0.4 (−0.5 to −0.3)
Fish	2.8 (2.5–3.1)	4.0 (3.9–4.2)	−0.5 (−0.6 to −0.4)
Eggs	1.4 (1.2–1.6)	2.1 (2.0–2.2)	−0.4 (−0.5 to −0.5)
Vegetable and legumes	4.7 (4.4–4.9)	4.7 (4.6–4.8)	0.0 (−0.1 to –0.1)
Fruits	2.9 (2.6–3.1)	3.6 (3.5–3.8)	−0.3 (−0.4 to −0.2)
Bread (including pizza, pita and tortillas)	2.7 (2.4–2.9)	3.0 (2.8–3.1)	−0.2 (−0.3 to –0.0)
Pasta	1.6 (1.4–1.9)	1.7 (1.5–1.8)	−0.1 (−0.2–0.0)
Rice	4.3 (4.1–4.6)	4.5 (4.4–4.6)	−0.1 (−0.2 to −0.0)
Awara	1.7 (1.4–1.9)	1.1 (1.0–1.3)	0.2 (0.1–0.3)
Cereals (Maize, millet, guinea corn)	4.9 (4.6–5.1)	3.7 (3.5–3.9)	0.5 (0.4–0.6)
Tubers (Yam, Cassava)	2.7 (2.5–3.0)	3.3 (3.1–3.4)	−0.3 (−0.4 to −0.2)
Acha	0.4 (0.2–0.5)	0.3 (0.2–0.4)	0.0 (−0.1 to –0.1)

*Note:* Data are presented as means of days individuals consumed food items with 95% CI. Effect sizes were using standardized mean difference (SMD) with 95% CI. Disorders of gut brain interactions (DGBI).

Individuals with any DGBI consumed less of their diet from animal sources (milk, meat, fish, and eggs), fruits, and some carbohydrate‐rich food items (pasta, bread, rice, and tubers) compared to those with no DGBI, although they had similar intakes of vegetables and legumes (common sources of FODMAP) and consumed more cereals (maize, millet, and guinea corn) compared to those with no DGBI.

## 4 Discussion

This study provides the first ever comprehensive national prevalence of DGBI based on the Rome IV criteria and associated factors in Nigeria. From the results of this study, the prevalence of having any DGBI in Nigeria was 23.5% with a tendency for higher prevalence among females and individuals aged ≥ 65 years. Functional dyspepsia was the most prevalent individual DGBI, while the highest prevalence of DGBI per GI anatomical region was bowel disorders. A north–south gradient pattern was observed with higher prevalences reported in the northern region compared to the southern region. Individuals with any DGBI were seen to have a higher burden of psychological, non‐GI somatic symptoms and healthcare utilization than those with no DGBI.

The prevalence rate for having any DGBI in Nigeria was lower than the global prevalence (internet‐based survey) of 40.3% [1], but among the highest in face‐to‐face survey countries where the overall prevalence was 20.7% [1]. As discussed in an earlier RFGES publication [18], differences in the data collection method may have contributed to the lower prevalence rates in face‐to‐face interview countries as anonymous internet‐based participants might have found it easier to express their symptoms than those who were interviewed face‐to‐face.

Data from this survey showed that the highest prevalence rate of any DGBI based on age group was seen in individuals ≥ 65 years and the lowest in those aged 18–39 years. This finding is consistent with what was observed for the overall face‐to‐face survey countries data [1] and as reported from Malaysia [27], a developing country like Nigeria that also used face‐to‐face household interviews to collect data but is different from most countries that used the internet survey [1] where the prevalence of any DGBI was highest in the younger age group 18–39 years. It is not clear why the pattern is different compared to the internet‐based countries, but the older age group could have been more likely to open up about their symptoms than the younger age groups due to shame. The predefined composition of the study population of 20% of adults ≥ 65 years is higher than the recent WHO reported figure of 4.1% of adults over 65 years in Nigeria, which may have resulted in overestimation of the national prevalence [8]. The reason for including 20% of adults ≥ 65 years in the survey was to ensure consistency in data collection across all countries [1, 18] in the RFGES. Our results from this survey also found that the prevalence of having any DGBI in Nigeria was higher in females than males, which is similar to what was reported in the face‐to‐face data of the global survey [18] and globally [1]. This result also further confirmed the female's preponderance in DGBI.

Our data on DGBI per GI anatomical region is the first to be reported for the Nigerian population and shows that bowel disorders were the most prevalent (17.6%) and esophageal disorders the least. There is no other available study in Nigeria or other African countries on DGBI per GI anatomical region for comparison. Functional dyspepsia had the highest prevalence (7.3%). Other prevalent DGBI were unspecified functional bowel disorder, proctalgia fugax, and functional constipation. Interestingly, functional constipation (4.2%) is not as prevalent as seen in other countries from global results of the face‐to‐face survey where a prevalence of 6.6% was reported [1]. Although functional dyspepsia appears to be the most prevalent DGBI in Nigeria, it is important to acknowledge that the *Helicobacter pylori* ( *H. pylori* ) status of the participants was not known, and this could have affected the prevalence of functional dyspepsia. This is important because a recent study [28] reported that prevalence of *H. pylori* infection in Nigeria ranges from 5.5% among university students to as high as 96% among symptomatic patients, with an overall national estimate between 30% and 87.7%. Due to lack of data regarding prevalence of DGBI among Nigerian adult population, it is difficult to make any comparison at this point and in addition to this, different diagnostic criteria (Rome II, III, IV and Manning) and settings (hospital, school/University and community) were used for data collection in previous studies in Nigeria. For example, a previous study [11] among adult patients attending a general outpatient clinic in north‐central Nigeria reported a high prevalence of 33% for IBS compared to our reported national prevalence of 3% and north‐central prevalence of 4.5%. The disparity in prevalence could partly be explained by the fact that the previous IBS study (Rome II criteria) included selected groups of patients attending a particular clinic, not from the community, and in addition our study used the more stringent Rome IV IBS diagnostic criteria resulting in lower prevalence rates. When a rural community in the same region was investigated (Rome II) [12], a prevalence of 31.6% was reported and this suggests that even within a rural community setting, IBS was prevalent despite the use of stringent Rome II criteria. However, a recent review of 20 studies from sub‐Saharan Africa (including 10 studies from Nigeria), the reported prevalence for IBS was 16% [29]. It is important to note that the stringent Rome II diagnostic criteria require a higher symptom frequency and a longer symptom duration, which results in a lower prevalence rate than Rome III based studies [30].

The northern region of Nigeria carried most of the burden of any DGBI with the highest prevalence of any DGBI seen in the north‐west region (39.6%) and lowest in the south‐east region (1.2%). This difference was also supported by higher psychological, non‐GI somatic symptoms and QOL scores. This may be explained by the socio‐cultural differences across these regions as DGBI distribution is known to be influenced by these factors [31, 32]. The northern region generally has a unified way of life and culture which is mostly different from the southern part and is relatively underdeveloped economically and medically [33]. Most people living in the northern region practice Islam as a religion and speak Hausa as a common language although other minority languages are spoken in the region, while in the southern regions Christianity is the major religion and several languages are spoken in the different states with Igbo and Yoruba languages being the predominant ones. It is not clear if the uniformity of the language in the northern region (Hausa) helped the interviewing staff administer the survey effectively and describe the questions to the respondents more clearly as the Hausa language has specific words to explain symptoms, possibly leading to higher prevalence rates. The educational level seen in the northern regions compared to the southern regions may also be a contributory factor in the higher prevalence in the northern region as higher levels of education have been associated with better health [34].

Most individuals with any DGBI (58.2%) lived in rural areas compared to those without (44.9%), although the unemployment rate was high in both groups. This may indicate that the poor living conditions commonly associated with rural areas might contribute to the higher prevalence rate for any DGBI in Nigeria compared to the urban areas. This finding is supported by earlier studies that indicated that lower socioeconomic class is a risk factor for upper and lower GI symptoms [35]. It is not clear if the type of religious belief the individual has could play a role in the prevalence of DGBI as more than half of the individuals with any DGBI were Christians and about 40% Muslims.

The results of dietary intake patterns showed that individuals with any DGBI had generally lower intakes from animal sources and fruits, which are relatively more expensive and associated with urban lifestyle than individuals with no DGBI. It may be viewed as surprising that individuals with DGBI appear to consume less fruit (rich in FODMAPs) than individuals with no DGBI as it is generally known that FODMAPs intake is associated with worsening of DGBI symptoms [36]. However, this association might partly be an effect of individuals with a DGBI reducing intake of fruits as a measure to reduce symptoms. Our findings may suggest that other fiber‐rich foods are driving the symptom pattern in the Nigerian population because we saw in this study that individuals with any DGBI also appear to consume more cereal (maize, millet, and guinea corn) than those with no DGBI who consumed more refined carbohydrates (bread, pasta). Cereal foods are considered staple in Nigeria especially in the northern region where the highest burden of DGBI is observed. This supports the role of environmental factors like diet and living conditions in the development of DGBI, which are known to be affected by socio‐cultural practices [37, 38]. However, our findings must be interpreted with caution as the data are cross‐sectional and do not establish a causal relationship between lifestyle factors and prevalence of DGBI.

Individuals with any DGBI in Nigeria have a higher burden of psychological symptoms (anxiety and depression), non‐GI somatic symptoms, work productivity and activity impairment than those individuals with no DGBI in Nigeria, as has been found in other countries globally [1, 39]. Although no previous study investigated the relationship of having any DGBI with psychological symptoms, one study in Nigeria reported a significant relationship between IBS and depression among rural community dwellers [12]. Having any DGBI also appears to be linked to increased healthcare utilization and lower years of education in the Nigerian population as reported in other countries as well [27, 40]. Although having an organic GI disease was a reason to exclude participants from having any DGBI in this survey, most of these diseases (coeliac disease and IBD) are linked to western countries. In tropical countries like Nigeria, *H. pylori* , tropical sprue, intestinal protozoal infections (giardiasis, cryptosporidium), worm infections (ascariasis and strongyloidiasis), intestinal tuberculosis and amoebiasis are common. These diseases can present with features of DGBI like chronic abdominal pain, bloating and diarrhea. For example, post‐infection IBS (PI‐IBS) has been reported to be linked to prior infectious enteritis in 4%–36% [41], 10% [42] and 10.5% [43]. Due to the high burden of gastrointestinal infections in Nigeria [44, 45] like most tropical countries, it is likely that prior infectious enteritis may be driving the national prevalence of IBS and DGBI in general. Although we have observed in our study that 7.7% of individuals with any DBGI self‐reported GI infections immediately prior onset of their bothersome symptoms, future studies in Nigeria and the tropics in general should consider adding these diseases to the list of organic diseases to be excluded for relevant DGBI diagnosis.

A major strength of this study is that it provides the first ever comprehensive data on the national prevalence of DGBI in the adult population of Nigeria where all geopolitical zones of the country were equally represented and all 22 DGBI diagnoses were surveyed. This provides reference data for future studies and could help with national healthcare planning, considering how prevalent DGBI are and their strong impact especially in the northern regions. Another strength is that the participants' age group, sex, and urban vs. rural living conditions were predetermined which allowed for fair representation of the Nigerian population covering both poor and the rich populations. The use of face‐to‐face household interviews in this survey allows for scientifically random selection of participants and provides the opportunity for the interviewers to explain the questions better if asked to do so.

A limitation of this study was that it required many trained interviewers to administer the questionnaires, which can be expensive. There is also the increased risk of reticence to respond to questions of a sensitive, possibly intimate nature in face‐to‐face interviews. In addition, the role of interviewers in household surveys always needs to be considered [18], and we cannot exclude that this might have partly affected the large regional differences in our survey. Although a predefined age group, sex, and geopolitical region composition was used for the survey, the large number of participants excluded 486 (23%) in our analysis as described in our methods slightly influenced the regional and age‐specific group prevalences. Another limitation of the study is that data were not collected on the ethnicity of the participants and the states where they came from or lived in. This is important because ethnic and cultural differences may have contributed to the perception of symptoms, reporting behavior, and DGBI distribution. Nigeria is a multi‐ethnic country made up of 36 states from the six geopolitical zones where the healthcare system is co‐funded by the states and federal governments, and each state has its own healthcare plans and budget that could influence the access and delivery of healthcare in their respective states. Another limitation of this survey was that all the questionnaires were administered in English. At times, the interviewers had to ask questions in the participant's spoken language thereby making their job more difficult and the study methodology less uniform. The proportion of subjects where the local language was used is not available as it was not part of the study design and therefore studies in Nigeria and similar countries should collect such information during interviews. The larger proportion of older individuals in our survey relative to the general Nigerian population may have overestimated the overall country prevalence, although the lower prevalence in the southeast region probably reflects an underestimation of the real prevalence. Therefore, future demographic surveys involving Nigeria should consider adjusting the composition of the survey participants to match the national age distribution.

## 5 Conclusion

In this comprehensive national population‐based study, we demonstrated that DGBI are prevalent and impactful in the Nigerian adult population, in line with studies from other similar countries around the world that utilized the face‐to‐face survey. Functional dyspepsia is the most prevalent individual DGB and bowel disorders are the most prevalent DGBI per GI anatomical region. Nigeria's northern geographic region shows a higher burden of DGBI compared to the southern region. More studies are needed to further understand the distribution and impact of DGBI in Nigeria and other parts of the world where they are understudied.

## Author Contributions

A.I.A. designed the study, performed the statistical analysis of the data, interpreted the results, and wrote the manuscript. T.G., J.P.H., H.T., E.O., S.I.B., A.D.S. and M.S. contributed to the study design, interpreted the results, and revised the manuscript. M.D., P.D., M.O., D.N., P.O., A.M., K.Z., C.O., A.A., O.S.P. and E.O. contributed to the study design for Nigeria, data acquisition, analysis, and interpretation. O.S.P., A.D.S., S.I.B. and M.S. were involved in the conception and implementation of the original study from which the current data were extracted. All authors approved the final version of the article.

## Funding

The authors have nothing to report.

## Conflicts of Interest

M.S. has received grant support from BioGaia; personal fees from Tillotts, Takeda, Biocodex, Danone, Sanofi, AlfaSigma, Abbvie, Janssen Immunology, Pfizer, BioGaia, Renapharma, Mayoly, and Bromatech. O.S.P. has received a research contract from the Rome Foundation during the conduct of the study and received a research contract from Glycom A/S and Royal DSM and personal fees from metaMe Health and iHealth. H.T. is an Advisory board member/consultant: Medifactia, Dr. Falk Pharma GmbH, Viatris, PRO.MED.CS; and Speakers bureau: Enterra Medical, Viatris. Other authors declared no conflicts of interest.

## Data Availability

The data that support the findings of this study are available on request from the corresponding author. The data are not publicly available due to privacy or ethical restrictions.
